# Low-intensity ultrasound enhances the anticancer activity of cetuximab in human head and neck cancer cells

**DOI:** 10.3892/etm.2012.739

**Published:** 2012-10-10

**Authors:** TAKASHI MASUI, ICHIRO OTA, MASATOSHI KANNO, KATSUNARI YANE, HIROSHI HOSOI

**Affiliations:** 1 Departments of Otolaryngology-Head and Neck Surgery and; 2 Oncology Center, Nara Medical University;; 3 Department of Otolaryngology, Kinki University School of Medicine, Nara Hospital, Nara, Japan

**Keywords:** cetuximab, low-intensity ultrasound, apoptosis, head and neck cancer

## Abstract

The potential clinical use of ultrasound in inducing cell apoptosis and enhancing the effects of anticancer drugs in the treatment of cancers has previously been investigated. In this study, the combined effects of low-intensity ultrasound (LIU) and cetuximab, an anti-epidermal growth factor receptor (EGFR) antibody, on cell killing and induction of apoptosis in HSC-3 and HSC-4 head and neck cancer cells, and its mechanisms were investigated. Experiments were divided into 4 groups: non-treated (CNTRL), cetuximab-treated (CETU), ultrasound-treated (UST) and the combination of cetuximab and US-treated (COMB). Cell viability was assessed by trypan blue staining assay and induction of apoptosis was detected by fluorescein isothiocyanate (FITC)-Annexin V and propidium iodide (PI) staining assay at 24 h after cetuximab and/or US treatment. To elucidate the effect of cetuximab and US on EGFR signaling and apoptosis in head and neck cancer cells after the treatments, the expression of EGFR, phospho-EGFR, and the activation of caspase-3 were evaluated with western blotting. More cell killing features were evident in the COMB group in HSC-3 and HSC-4 cells compared with the other groups. No differences in EGFR expression among the CETU, UST and COMB groups was observed, while the expression of phospho-EGFR in the CETU group was downregulated compared with that in the CNTRL group. Phospho-EGFR expression was much more downregulated in the COMB group compared with that in the other groups. In addition, the activation of caspase-3 in the UST group was upregulated compared with that in the CNTRL group. Caspase-3 activation was much more upregulated in the COMB group than that in the other groups. These data indicated that LIU was able to enhance the anticancer effect of cetuximab in HSC-3 and HSC-4 head and neck cancer cells.

## Introduction

Head and neck squamous cell carcinoma (HNSCC) is known for its rapid clinical progression and poor prognosis. The survival rates for many types of HNSCC have improved little over the past 40 years. Anti-monoclonal antibody is often used as a first-line and primary treatment of malignancies, such as malignant lymphomas and breast cancer. Cetuximab, an anti-epidermal growth factor receptor (EGFR) antibody, is the only EGFR inhibitor approved in HNSCC. Several combined methods with cetuximab, such as irradiation or many types of anticancer drugs, have been applied to enhance the effect of treatment. However, the efficacy of such treatment is inadequate. Therefore, new therapeutic methods should be developed to improve the survival rate.

In non-surgical treatment, induction of apoptosis is a preferred mode of killing cancer cells with fewer side effects and immune reactions (1). Apoptosis is a physiologic process that contributes to the homeostasis of multi-cellular organisms and maintains the balance between cell proliferation and cell death (2), as a cell response to stress stimuli, such as exposure to chemotherapeutic drugs, oxidative stress, free radicals, X-rays, ultraviolet radiation, shear stress and ultra-sound (US) irradiation.

Since the biological effect of US was first reported in 1927, numerous reports concerning the application of US have been published. The mechanisms of US-induced apoptosis on physical and biochemical levels have been discussed in several studies (3–7). In addition, US irradiation can be divided into low-intensity ultrasound (LIU) and high-intensity focused ultrasound (HIFU) depending on the intensity. LIU has a great potential in apoptosis therapy for cancer (8), while HIFU can thermally ablate tissues via hyperthermia in various types of cancer (9,10). LIU has been subjected to numerous studies to evaluate its biological effects and their possible application in therapeutic strategies in cancer treatment. Thes]e outcomes include the enhancement of cellular uptake of drugs (11) and the induction of apoptosis and cell killing (12,13). Besides, it is likely that the selectivity of LIU for cancer cells is higher than that for normal cells (14).

The combined effects of US and several anticancer drugs, such as (DOX) and Cisplatin (CDDP) have often been reported (15,16). Thus, sonication by US may also be considered as a candidate for the enhancement of anticancer drugs. However, few reports associated with anti-monoclonal antibody are available. We hypothesized that US has the potential to become an enhancer of cetuximab owing to its effect of changing cell membrane structure and making cancer cells more sensitive to cetuximab as well as rituximab, as previously reported (17).

In the present study, we assessed the therapeutic potential of the combination of cetuximab and LIU *in vitro* and demonstrated that this combination induces the effect of tumor cell killing and increases the therapeutic efficacy in head and neck cancer HSC-3 and HSC-4 cells. In addition, we focused on EGFR signaling and apoptosis signaling through the mitochondria-caspase pathway to confirm cetuximab enhancement by US.

## Materials and methods

### Cell culture and drugs

Human HNSCC cells, HSC-3 and HSC-4, were employed in this study. The HSC-3 and HSC-4 cells, obtained from the Japanese Cancer Research Resource Bank (Tokyo, Japan), were cultured in DMEM (Invitrogen, Carlsbad, CA, USA) supplemented with 10% heat-inactivated fetal bovine serum (Invitrogen), 100 U/ml penicillin, and 100 mg/ml streptomycin (Gibco, Grand Island, NY, USA) at 37°C in 5% CO_2_. Cetuximab (Merck KgaA, Darmstadt, Germany) was dissolved in phosphate-buffered saline (PBS) at a concentration of 100 nM and then stored at 4°C until use.

### US apparatus and intensity

A 1.0 MHz ultrasonic generator (KUS-2S; ITO Ultrasonic Co., Ltd., Tokyo, Japan) with a fixed duty factor of 50% and with 1 Hz pulse repetition frequency (PRF) was used. The sonication was conducted at an intensity of 0.5 W/cm^2^ (= output intensity of 0.38 W/cm^2^) and exposure time of 1 min. The intensity and time were used in all of the sonication experiments.

**Figure 1:**
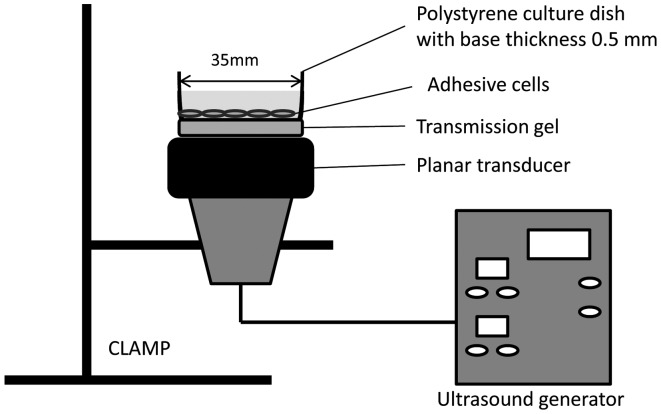
Ultrasound exposure system.

To keep the transducer-facing directory upward for the sonication procedure, the transducer, with a diameter of 2.7 cm, was fixed with a clamp attached to a metal stand. A 3.5-cm culture dish was placed on the center of the transducer with intermediate gel (Fig. 1).

### Experimental protocol

The experimental groups for this study were: i) non-treated (CNTRL), ii) cetuximab-treated (CETU), iii) US-treated (UST) and iv) the combination of cetuximab and US-treated (COMB). Cells (1×10^6^) in 1.5 ml medium were seeded in a 3.5-cm dish and incubated at 37°C for 12 h. At 30 min prior to sonication another 1.5 ml of fresh medium, with or without cetuximab at the final concentration of 100 nM, was added to each dish to avoid cavitation attenuation due to the high concentration of carbon dioxide accumulated in the dish, as previously described (18,19). Following the treatment, the cells were subjected to different analyses.

### Measurement of cell viability

Cell viability was assessed by trypan blue staining assay at 24 h after cetuximab and US, as previously described (6). The number of cells was counted using a hemocytometer to estimate the viability. The cell viability was calculated as the number of viable cells in the treated group/the number of viable cells in the non-treated group. Each measurement was repeated three times independently.

### Detection of apoptosis

Apoptosis was assessed by fluorescein isothiocyanate (FITC)-Annexin V and propidium iodide (PI) staining assay according to the manufacturer’s instructions (Roche Diagnostic GmbH, Penzberg, Germany). Following the treatment of cetuximab and US, the cells were incubated at 37.0°C for 24 h. The cells were then washed with cold PBS, stained with FITC-Annexin V/PI for 10 min and observed under a fluorescent microscope for typical morphological changes characteristic of apoptosis.

FITC-Annexin V/PI staining assay was performed to detect the phosphatidylserine expression using FITC-Annexin V labeling as an endpoint indicator of early apoptosis, and the PI uptake as an indicator of necrosis. Annexin V-positive and PI-negative cells were considered to be early apoptosis, while Annexin V-positive and PI-positive cells were considered to be secondary necrosis (6,15). Apoptosis was calculated as the number of apoptotic cells/the number of total cells in each group. Each measurement was repeated three times independently.

### Antibodies and immunoblot analysis

EGFR, phospho-EGFR, and activation of caspase-3 signaling on HSC-3 cells after the treatment were also evaluated with western blotting. Cells were cultured in 3.5-cm dishes for 6 h after the treatment, respectively. The cells were collected and frozen in 100 *μ*l RIPA buffer, and stored in a −30°C ultra low temperature freezer. Briefly, total protein extracts were prepared according to the freeze-thaw lysis method (20) and protein concentrations were measured with the bovine serum albumin (BSA) protein assay. Extract samples containing 20 *μ*g of protein were then separated by sodium dodecylsulfate-polyacrylamide gel electrophoresis (SDS-PAGE) and transferred to polyvinylidene difluoride membranes. The membranes were incubated with anti-EGFR, anti-phospho-EGFR, and anti-caspase-3 antibody [Cell Signaling Technology, Danvers, MA, USA; diluted 1:2000, 1:1000 and 1:1000 in PBS with Tween-20 (PBST) respectively] at 4°C overnight, and then with peroxidase-conjugated secondary anti-rabbit or goat immunoglobulin G (IgG) (Cell Signaling Technology; diluted 1:1000 in PBST) for 1 h. After rinsing in PBST (4 times, 5 min each), immuno detection was performed using an enhanced chemiluminescence (ECL) western blot analysis detection reagants and analysis system. The membranes were subsequently exposed to X-ray film, as previously described (21).

### Statistical analysis

Data were presented as the mean ± standard deviation (SD). Significant differences between groups were assessed using two-way factorial analysis of variance (ANOVA). P<0.01 was considered to be a statistically significant difference.

## Results

### Enhancement of cetuximab-induced cell killing by US

**Figure 2:**
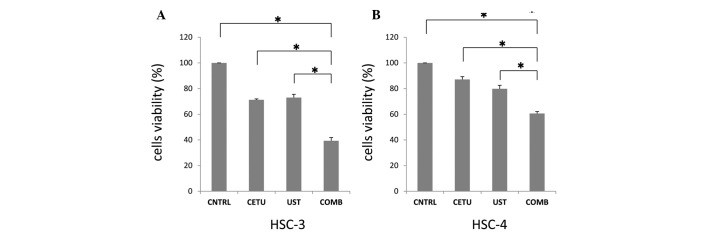
Enhancement of cetuximab-induced cell killing by ultrasound (US). Cetuximab was used at a concentration of 100 nM and sonication was performed at intensities of 0.5 W/cm^2^ for 1 min. Viability was determined by trypan blue staining assay 24 h after treatment in (A) HSC-3 cells and (B) HSC-4 cells. The data indicate the mean ± standard deviation (SD) and values are representative of more than three different experiments. Values (*) assessed as synergy by two-way factorial analysis of variance (ANOVA); ^*^P<0.01. COMB showed a synergistic enhancement in cell killing compared with the other treatments. CNTRL, non-treated; CETU, cetuximab-treated; UST, ultrasound-treated; COMB, combination of cetuximab and US-treated.

Cell viability was assessed by trypan blue staining assay at 24 h after cetuximab and US. The cell viabilities of HSC-3 cells (Fig. 2A) and HSC-4 cells (Fig. 2B) in the CETU group were 71.2 and 87.1% compared to those of the CNTRL group, respectively. The cell viabilities in the UST group were 73.0 and 79.8%, while those in the COMB group decreased to 39.3 and 60.6%, respectively, thus showing a synergistic enhancement of cetuximab-induced cell killing by US.

### Enhancement of cetuximab-induced apoptosis by US

**Figure 3:**
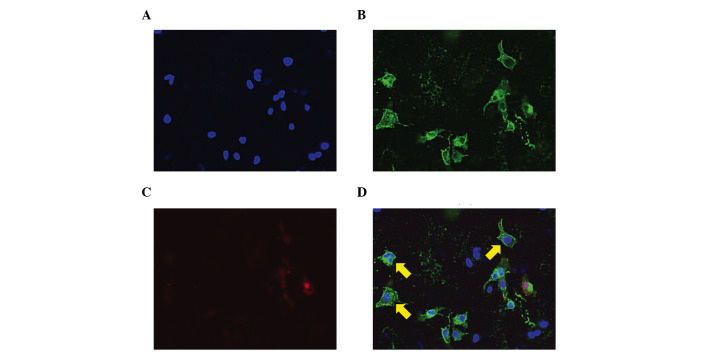
Transmission light microscope image of combined treatment in the COMB group of HSC-3 cells. (A), DAPI; (B), Annexin V; (C), propidium iodide (PI); (D) merged images. Fluorescein isothiocyanate (FITC)-Annexin V-positive and PI-negative cells were considered to be early apoptosis (arrows), while FITC-Annexin V-positive and PI-positive cells were considered to be secondary necrosis.

**Figure 4:**
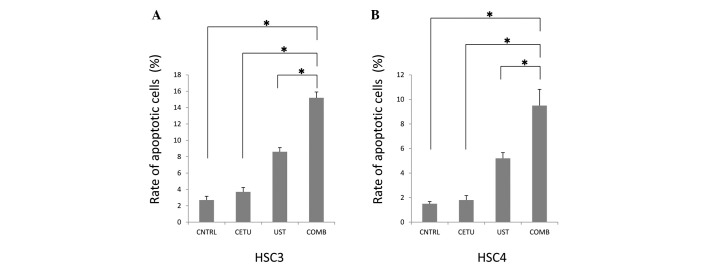
Enhancement of cetuximab-induced apoptosis by ultrasound (US). Induction of apoptosis was assessed by FITC-Annexin V/PI assay 24 h after treatment in (A) HSC-3 cells and (B) HSC-4 cells. The data indicate the mean ± standard deviation (SD) and values are representative of more than three different experiments performed. Values (^*^) assessed as synergy by two-way factorial analysis of variance (ANOVA); ^*^P<0.01. Significantly more apoptosis was observed in the COMB group compared with the other groups. CNTRL, non-treated; CETU, cetuximab-treated; UST, ultrasound-treated; COMB, combination of cetuximab and US-treated.

In order to estimate the enhancement of cell killing, the induction of apoptosis was assessed by FITC-Annexin V/PI staining assay (Figs. 3 and 4). FITC-Annexin V/PI staining assay was performed to detect the phosphatidylserine expression on the cell membrane as an endpoint of early apoptosis, showing the exact percentage of cells in viable cells (Annexin V−/PI−), early apoptotic cells (Annexin V+/PI−) and secondary necrosis (Annexin V+/PI+) under a fluorescent microscope. Fig. 3 shows (A) DAPI, (B) Annexin V, (C) PI and (D) merged images of the combined treatment on HSC-3 cells, respectively. The rates of apoptotic features of HSC-3 cells (Fig. 4A) and HSC-4 cells (Fig. 4B) in the CNTRL group were 2.7 and 1.5%, respectively, while in the CETU group, they were 3.7 and 1.8%, respectively. No statistically significant difference was observed between these two groups. In the UST group, the apoptotic features were 8.6 and 5.2%, respectively, while those in the COMB group were 15.2 and 9.5%, respectively. These data suggest that US induced apoptosis in these cells and that the combination of US and cetuximab enhanced apoptosis synergistically.

### Effects of EGFR signaling and apoptosis following the treatments

**Figure 5:**
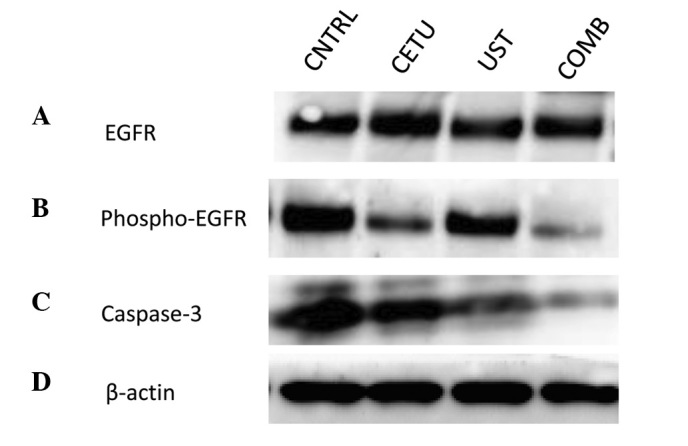
The expression of EGFR, phospho-EGFR, and activation of caspase-3 signaling on HSC-3 cells after the treatment with western blot analysis. (A), EGFR; (B), phosphor-EGFR; (C), caspase-3; (D), β-actin. There were no differences of the expression of EGFR among the CETU, UST and COMB groups, while the expression of phospho-EGFR was down-regulated and moreover the cleavage of caspase-3 was upregulated in the COMB group compared with the other groups. CNTRL, non-treated; CETU, cetuximab-treated; UST, US-treated; COMB, combination of cetuximab and US-treated

To elucidate the effects of EGFR signaling and apoptosis on HSC-3 cells after the treatments were administered, the expression of EGFR, phospho-EGFR, and the activation of caspase-3 were evaluated using western blotting (Fig. 5). No statistically significant differences in EGFR expression among the CETU, UST and COMB groups was observed, while the expression of phospho-EGFR was much more downregulated in the COMB group compared with the remaining groups, although phospho-EGFR was downregulated in the CETU group. These data suggest that US induced the inhibition of EGFR-signaling pathway by cetuximab. To elucidate the induction of apoptosis, the activation of caspase-3 was estimated. The activation, the cleavage of caspase-3, in the UST group was upregulated compared with that in the CNTRL group. Additionally, caspase-3 activation was much more upregulated in the COMB group compared with that in the remaining groups, suggesting that US induced apoptosis through the activation of caspase-3 by cetuximab.

## Discussion

In recent years, there has been a focus on the potential of LIU as well as HIFU in clinical applications. These outcomes include the enhancement of cellular uptake of drugs and the induction of cell killing or apoptosis (15,16,22). LIU primarily inhibits cell proliferation through its heating, cavitation and mechanical effect. Such investigations indicate that the biological effectiveness of US is linked to the acoustic conditions (23). In general, the impulsive pressures generated by the collapse of cavitation bubbles associated with US may permeabilize the plasma membrane of neighboring cells (24–27). This process allows exogenous molecules to enter the cells resulting in a biological response (27–32). Following the application of US, the cell membrane surface becomes rough and reversible within 24 h after US exposure (33,34). Tumor cells are more sensitive to US compared with normal cells and there is a threshold dose that effectively treats tumor cells with little effect on normal cells (4). In addition, the duration time and dose of LIU depend on tumor cell types. Generally, to avoid unexpected immune responses *in vivo* resulting from cell lysis induced by focused US at higher intensity, the dose of LIU is should be 0.5 W/cm^2^ below. In a previous report, there was a significant effect of US-induced apoptosis at more than 0.3 W/cm^2^ for 1 min, but not at less than 0.2 W/cm^2^ compared with non-treatment (15), suggesting that the threshold of the US effect is between 0.2 and 0.3 W/cm^2^. It has been shown that LIU, such as 1 MHz pulsed US at an intensity of 0.3 or 0.5 W/m^2^ for 1 min, is effective enough to induce apoptosis for cancer cells (15). Therefore, we utilized this dose of 0.5 W/cm^2^ for 1 min to obtain more effective apoptosis with minimal lysis for the HSC-3 and HSC-4 cells used in this study.

Furthermore, the possible mechanisms of US-induced apoptosis reportedly involve its cavitations (6,7,12,29,35). Apoptosis is known to require the activation of caspases, a group of enzymes involved in apoptotic cascade events (36). Apoptotic stimulus is capable of activating apoptosis-related proteins to enter mitochondria inducing the mitochondrial membrane to form pores to release molecules into the cytosol, such as cytochrome c. The released molecules activate caspase-9, which cleaves procaspase-3 to caspase-3, inducing apoptosis (37,38). Among these apoptosis-related proteins, caspase-3 plays a crucial role in apoptosis through the mitochondria-caspase pathway, and its activation is often used as a marker of apoptosis. Previous studies have shown that on a molecular level, several proteins, including p53, Bid and Bcl-2, were identified as responding to US irradiation, suggesting that mitochondrial membrane permeabilization, or pore formation, was involved in LIU-induced apoptosis (22,39). Additionally, disruption of the mitochondrial transmembrane potential, cytochrome c release, and caspase activation were also observed after US treatment (30). Thus, evidence suggests that US exposure is associated with cell apoptosis, which is mitochondria-caspase-dependent. This theory has provided a foundation for the clinical application of US. Consequently, numerous reports concerning the enhancement of anticancer drug by LIU are available. However, there have been few reports associated with anti-monoclonal antibody for cancer cells.

Cetuximab, a chimeric human:murine IgG1 monoclonal antibody against EGFR, is the most studied targeted therapy in HNSCC. It can be either inhibitory for cell growth or cytotoxic and can enhance the cytotoxic effect of chemotherapeutic drugs or ionizing radiation, resulting in cell apoptosis (40). Cetuximab is currently approved in combination with radiation therapy in locally advanced disease, as a single agent in platinum-refractory recurrent/metastatic disease, and in combination with platinum (carboplatin or CDDP) and 5-fluorouracil, as first-line therapy in recurrent/metastatic disease. However, response rates as a single agent have been less than satisfactory (13%) and of limited duration (2–3 months) (41). Thus, we suggest that US has the potential to become an enhancer of cetuximab owing to its effect of changing cell membrane structure and making cancer cells more sensitive to cetuximab as well as rituximab, as previously reported (17).

In the present study, we demonstrated that there were more tumor cell killing features in the COMB group than in the other groups and that the phosphorylation of EGFR was down-regulated, while the activation of caspase-3 was upregulated in the COMB group compared with that in the remaining groups. These data suggest that LIU was able to increase the induction rate of cetuximab in head and neck cancer cells, leading to enhancement of the effect of cetuximab in the COMB group. In addition, as previously reported, the activation of caspase-3 also increased when apoptosis was induced by US alone in cancer cells (22). These results suggest that an increase of the induction rate of cetuximab by US could subsequently activate caspase-cascade reaction more than US alone.

In summary, the present study has shown the synergistic enhancement of apoptosis induction by the combination of LIU and cetuximab in HNSCC cells. The mitochondria-caspase pathway is considered to be crucial in this process. These data suggest that LIU enhanced the anticancer effect of cetuximab in HSC-3 and HSC-4 cells of HNSCC. Although the precise involvement of the EGFR and caspase signaling modifications by cetuximab in the enhancement remains to be elucidated, they may be involved in the latent effect. The critical mechanisms remain to be further investigated. The majority of studies on US effects have been performed *in vitro* to evaluate clinical cancer therapies. However, it should be noted that the results of *in vitro* studies on the biological effects of therapeutic US cannot be translated to the same cells *in vivo*, even if the same US exposure is used. Although it is still too early to discuss its clinical efficiency, LIU may prove to be a valid treatment option for HNSCC.
